# Research on the Impact of Intercustomer Social Support on Customer Engagement Behaviors in Virtual Brand Communities

**DOI:** 10.3390/bs13010031

**Published:** 2022-12-29

**Authors:** Xuexin Li, Congcong Yang, Shulin Wang

**Affiliations:** 1 Business School, Faculty of Economics, Liaoning University, Shenyang 110000, China; 2 Sunwah International Business School, Faculty of Economics, Liaoning University, Shenyang 110000, China

**Keywords:** virtual brand communities, intercustomer social support, customer engagement behaviors, self-efficacy, interdependent self-construal

## Abstract

Customer engagement behavior is a key factor in promoting the sustainable growth of virtual brand communities. Despite the extensive research on the antecedents of customer engagement behaviors, the influence of intercustomer social support remains a neglected area in the context of virtual brand communities. Based on a total of 293 valid questionnaires collected via an online survey, a structural equation model and hierarchical regression analysis are used to examine the effects of intercustomer social support (informational/emotional support) on customer engagement behaviors (customer-oriented/community-oriented engagement behaviors) in virtual brand communities, as well as consider the mediating role of self-efficacy and the moderating role of interdependent self-construal in the above relationships. The empirical finding shows that informational/emotional support significantly affects customer-oriented and community-oriented engagement behaviors. Self-efficacy plays a mediating role in the relationship between informational/emotional support and customer-oriented/community-oriented engagement behaviors. Interdependent self-construal positively moderates the relationship between informational/emotional support and customer-oriented engagement behaviors and positively moderates the relationship between informational support and community-oriented engagement behaviors. This article provides a more comprehensive understanding of the relationships between intercustomer social support and customer engagement behaviors in the context of virtual brand communities, and improves the existing customer engagement behaviors management practices that are beneficial for the companies.

## 1. Introduction

Virtual brand communities (VBCs) are specialized, non-geographically bound online communities that are based on structured social relations [1,2] where people can communicate with each other and share their experiences and attitudes toward a given brand [3,4]. The increase in virtual brand communities will, to some extent, lead to a decline in the number of physical stores, which can help a country optimize the construction of urban land and reduce the urban noise caused by physical businesses [5,6]. Moreover, these communities contribute to companies by promoting brand loyalty among customers and developing long-term customer–brand relationships [7], and they help decrease the management costs of these companies by reducing the shops’ high rent and the investment in the shops’ environment [8]. Therefore, VBCs have become a strategic tool for brands in their management of customer relationships [9,10]. Many companies have established their VBCs and have made substantial investments in them to improve their engagement with customers [11]. However, a lack of motivation for user participation and low user activity are problems that are currently faced by VBCs [12]. More than half of the companies that sponsor virtual communities fail to realize their value; managers are faced with the challenge of maintaining customer engagement and enhancing community sustainability [13,14].

Making engaged customers active partners in the value creation and innovation processes is an important strategy to succeed in today’s highly competitive business environment [15]. Customer engagement places the customer’s behavior and attitude at the center of the marketing actions for strategic marketing as well as strategic management purposes [15]. Managers believe that engaging with their customers can create significant value in virtual communities through certain actions, such as word-of-mouth (WOM), referrals, offering feedback to a community sponsor, and helping others [14]. Existing studies have referred to these actions as customer engagement behaviors (CEBs); these are understood as non-transaction-bound, voluntary, and discretionary customer behaviors resulting from motivational drivers [16,17]. Engagement behaviors have been found to enhance the value of the virtual community [18,19,20]. Firms can ensure profitable growth and sustainable competitive advantages through CEBs [21,22]. Previous studies have emphasized the importance of studying the drivers of CEBs, especially the customer-based drivers [15,18]. Moreover, customers often join VBCs to seek support from other customers when they encounter product problems [23]. Within intercustomer social connections, customers enhance their unique experience with the service, which leads to positive outcomes for the organization [24,25]. A study in a nursing home showed that individuals who receive social support from other customers report an intent to perform CEBs [17]. Differently from traditional service organizations, the social support that individuals receive from other customers in VBCs is intangible because of the virtuality of the network [23]. Those intangible assets impact a firm’s financial performance by providing a market-based asset [25,26]. There remains a lack of understanding of how intercustomer social support affects CEBs in the context of VBCs. Thus, to address this gap, this article extends intercustomer social support to the context of VBCs to explore its effects on CEBs.

It is noteworthy that the role of self-efficacy and interdependent self-construal cannot be ignored in the relationship between intercustomer social support and CEBs. Specifically, self-efficacy—interpreted as the belief in one’s own ability to successfully execute actions—is an important antecedent of customer behavior [27,28]. According to the social cognitive theory, social support can promote self-efficacy by building knowledge and emotional resources among individuals and, accordingly, influencing their behaviors [27,29]. Bravo et al. [28] found that self-efficacy has positive effects on CEBs, such as word-of-mouth (WOM), creating content, and providing feedback. Therefore, this study examines the mediating role of self-efficacy in the relationship between intercustomer social support and CEBs. In addition, self-construal is related to cognition, emotion, motivation, and behavior [30,31]. People tend to show interdependent self-construal due to the technical features of social networking sites [32]. Compared with other forms of self-construal, interdependent self-construal can better predict online behavior [33,34,35]. Therefore, this article focuses on interdependent self-construal. Furthermore, interdependent self-construal customers at different levels also have differences in their understanding ability and cognition toward group resources. People who show a high level of interdependent self-construal perceive more supportive social relationships [36]. Thus, this study examines the moderating role of interdependent self-construal in the relationship between intercustomer social support and CEBs.

This study aims to explore the effects of intercustomer social support (informational/emotional support) on CEBs (community-oriented/customer-oriented engagement behavior) in the context of VBCs. Furthermore, this study examines the mediating role of self-efficacy and the moderating role of interdependent self-construal in the above relationships.

Overall, this study contributes to the literature by extending the relationship between intercustomer social support and CEBs to the context of VBCs. Moreover, drawing on the social cognitive theory, the influence paths of the above relationships are determined by examining the mediating role of self-efficacy. In addition, drawing on the self-construal theory, this study expands the boundary conditions of the effects of intercustomer social support on CEBs by examining the moderating role of interdependent self-construal, which also contributes to the literature. The results of this research add to a more comprehensive understanding of the relationships between intercustomer social support and CEBs in the context of VBCs, which have values for companies to manage customer relationships and successfully operate VBCs.

## 2. Theory and Hypotheses

### 2.1. Intercustomer Social Support and CEBs

Intercustomer social support is a form of customer-to-customer social support [37]. Black et al. [25] define intercustomer support as “the customers’ perception of the resources they receive from other customers within the service setting that result in feelings of belonging and enrich the service experience”. Following Black et al. [25], we define intercustomer social support as the support that customers experience when they receive advice, help, and concern from other customers [38,39,40]. Customers can obtain two types of social support from other customers: emotional support and instrumental support [41]. Furthermore, instrumental support can be categorized as either informational support or material support [42]. Emotional support and informational support comprise the major mechanisms of support in social interactions and are the main targets of individual social support in virtual communities [43]. This study agrees with the above points of view and regards emotional support and informational support as two dimensions of intercustomer social support. Informational support refers to messages—in the form of recommendations, advice, or knowledge—received from other customers, and emotional support refers to emotional messages such as caring, understanding, or empathy [43,44].

CEBs are non-transactional behavioral manifestations with a “brand or firm focus, resulting from motivational drivers” [16]. CEBs can benefit the performance of a firm in two ways: through interactions between firms and their employees, such as providing feedback for employees, cooperating with employees, and complying with organizational rules and procedures; through interactions with other customers, such as helping other customers by writing online reviews and spreading positive a WOM [17]. In line with a study by Verleye et al. [17], Han et al. [45] divided the CEBs in the virtual community into two dimensions: community-oriented engagement behavior and customer-oriented engagement behavior. Based on previous studies, this study argues that community-oriented engagement behavior and customer-oriented engagement behavior are two dimensions of CEBs [45]. Specifically, community-oriented engagement behavior refers to the CEBs of customers toward their community, including feedback, cooperation, and compliance [17,45]. Customer-oriented engagement behavior refers to the CEBs of customers toward other customers, including providing help and engaging in positive WOM [17,45].

Reliable and friendly informational and emotional communication have positive impacts on customers’ psychological states, leading to voluntary engagement with and contribution to VBCs [46,47,48]. For example, in an online community, members who receive social support from other members have a high level of satisfaction with the community [49]. Customers with high levels of satisfaction provide feedback to the firm, make recommendations [23], and help the community improve its services by cooperating with it [22]. In addition, Verleye et al. [17] point out that social support from other customers positively affects customers’ role readiness. Based on the role theory, customers’ role readiness depends on their level of confidence and the knowledge they possess in interacting with an organization [17]. High role readiness among customers enhances their compliance with organizational rules and other CEBs, such as cooperation and feedback [17,50]. Therefore, informational support and emotional support from other customers in the VBCs encourage customers to perform community-oriented engagement behaviors.

Based on the above analysis, this article proposes the following hypotheses:

**H1.**  *(a) Informational support and (b) emotional support have positive effects on community-oriented engagement behaviors.*

Informational support can increase the informational resources available to customers; meanwhile, emotional support can deepen customers’ emotional connections and sense of partnership within the VBCs. When individuals gain benefits from the social support of others, they will repay that support to others [51]. That is, in the VBCs, when customers receive informational support and emotional support from other customers, they reciprocate this support by, for example, helping other customers. Moreover, informational and emotional support generates an interactive motivation among customers, which encourages the customers to perform customer-oriented engagement behaviors, such as share shopping information and recommendations [43]. Therefore, customers who receive informational and emotional support from other customers in the VBCs will perform customer-oriented engagement behavior out of reciprocal motivation.

Based on the above analysis, this article proposes the following hypotheses:

**H2.**  *(a) Informational support and (b) emotional support have positive effects on customer-oriented engagement behaviors.*

### 2.2. Mediation of Self-Efficacy

Self-efficacy refers to the belief that one can successfully execute actions, and it plays an important role in the motivations and behaviors of individuals [27]. Liu et al. [52] argue that, in social media brand communities, self-efficacy reflects the degree to which consumers believe that they have relevant knowledge resources to engage in role-playing. The acquisition and formation of self-efficacy can be attributed to the power of four factors: performance accomplishments, vicarious experience, verbal persuasion, and emotional arousal [27]. Among these, informational support and emotional support can be viewed as vicarious experience and emotional arousal factors, respectively. As a factor of vicarious experience, informational support—especially informational support involving the experiences of others—can help individuals master more information resources by providing guidance and advice [53,54]. Subsequently, informational support can strengthen the self-efficacy of individuals [29]. As a factor of emotional arousal, emotional support can help relieve individuals’ negative emotional arousal and keep individuals in a positive psychological state, which helps to improve self-efficacy [27,29].

Based on the social cognitive theory, self-efficacy can cause or activate an individual’s different environmental reactions, and then strongly guide and dominate an individual’s behavior [27,55]. The higher the level of self-efficacy, the more likely the individuals are to perform certain behaviors and be optimistic about their ability to control threatening situations [27]. Informational support and emotional support from other customers can increase individuals’ brand knowledge and community happiness by reducing stress, tension, depression, and the fear of making mistakes [40,41]. This support from others can also strengthen an individual’s sense of control and the feeling of being cared for—stimulating positive emotional states such as self-confidence and social integration—which are conducive to improving self-efficacy and consequently have positive effects on customers’ behavior intentions [40,41]. Furthermore, Wang and Fesenmaier [56] demonstrated that self-efficacy has a vital influence among customers contributing to the online travel community. Bravo et al. [28] reveal that people who have a high level of self-efficacy will actively engage in CEBs, such as spreading positive WOM and providing feedback.

Based on the literature discussed here, this article proposes the following hypotheses:

**H3.**  *Self-efficacy mediates the effects of (a) informational support on community-oriented engagement behaviors, (b) emotional support on community-oriented engagement behaviors, (c) informational support on customer-oriented engagement behaviors, and (d) emotional support on customer-oriented engagement behaviors.*

### 2.3. Moderation of Interdependent Self-Construal

According to the self-construal theory, self-construal is the feeling of an individual in relation to others; it reflects the extent to which people define themselves according to their connectivity within groups and collectives [33]. The basic springboard is that people from different cultures have fundamentally different perspectives on the relationship between the self and others; Westerners who advocate for an individualistic culture are more likely to exhibit independent self-construal, while Easterners who advocate for a collectivistic culture are more likely to exhibit interdependent self-construal [30,57,58]. Individuals with an independent self-construal emphasize the difference between the self and others; the self-representation associated with this mindset mostly involves personal traits, abilities, and preferences [59]. In contrast, individuals with an interdependent self-construal focus on the relationship between themselves and others, and their self-representation is mostly based on interpersonal communication [59]. In addition to cultural factors, a special context also activates a certain sense of self within individuals [60]. For example, the technical characteristics of social networking sites predispose individuals to exhibit interdependent self-construal [33]. In addition, people define themselves more in terms of their collective self when they can benefit from group membership [61]. Compared with independent self-construal, interdependent self-construal can better predict online behaviors [33,34,35].

An interdependent self-construal is interpreted as the extent to which individuals include relationships in their self-definition [30,62,63]. Individuals with an interdependent self-construal view themselves as part of a group, and they value and rely on social connections [58]. Community-oriented engagement behavior can satisfy customers’ willingness to be part of the community. Some studies have pointed out that people with an interdependent self-construal tend to comply with situational norms [30,64]. Moreover, in the VBCs, customers have the same opportunity to receive social support from other customers. When individuals receive informational and emotional support in a fair procedure, individuals with high levels of interdependent self-construal perform more cooperative behaviors [65]. Moreover, compared with low-level interdependent self-construal, individuals with high levels of interdependent self-construal easily perceive various social supports and have a high degree of acquisition and recognition of external resources [66]. Therefore, compared with customers with low levels of interdependent self-construal, customers with high levels of interdependent self-construal perform more community-oriented engagement behaviors when they receive informational and emotional, social support from other customers in the VBCs.

Based on the above analysis, this article proposes the following hypotheses:

**H4.**  *Interdependent self-construal positively moderates the relationships between (a) informational support and community-oriented engagement behaviors, and (b) emotional support and community-oriented engagement behaviors.*

Meanwhile, customer-oriented engagement behavior can satisfy their willingness to maintain group contact and harmonious relationships. To maintain a harmonious relationship with others, individuals with an interdependent self-construal have more social and subsidiary motivations [67]. The supportive characteristics of intercustomer social support exactly fit the characteristics of interdependent self-construal because the psychological projection mechanism of interdependent self-construal is an injective projection of “self-determination by others” [68]. Hofmann et al. [69] point out that individuals with interdependence engage more in word-of-mouth. Holland et al. [70] argue that a strong interdependent self-construal can enhance cooperative and supportive behaviors among customers. Furthermore, people with an interdependent self-construal pay more attention to their social responsibility, social roles, and harmonious relationships in the group and are highly motivated to share with others [71]. Therefore, when customers receive informational and emotional, social support from other customers, customers with high levels of interdependent self-construal perform more customer-oriented engagement behaviors.

Based on the above analysis, this article proposes the following hypotheses:

**H5.**  *Interdependent self-construal positively moderates the relationships between (a) informational support and customer-oriented engagement behaviors, and (b) emotional support and customer-oriented engagement behaviors.*

**Figure 1:**
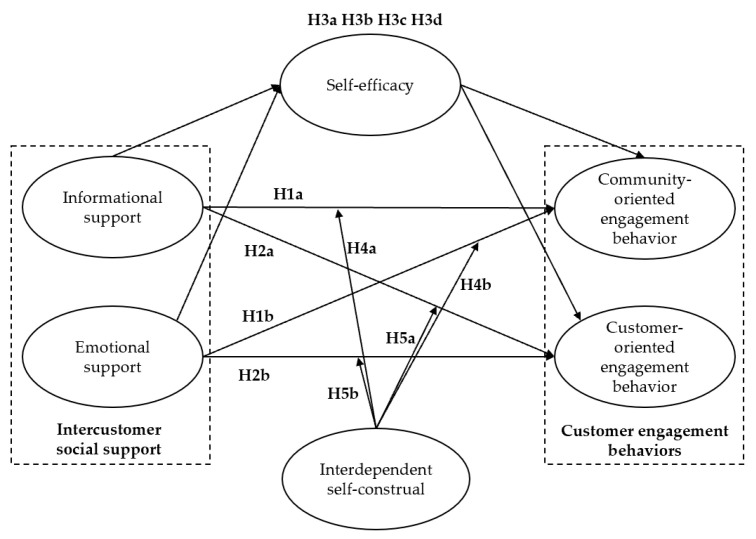
Research model.

Based on the above discussion, this study develops a model that integrates intercustomer social support (informational support and emotional support), CEBs (community-oriented engagement and customer-oriented engagement behaviors), self-efficacy, and interdependent self-construal, as shown in Figure 1.

## 3. Methods

### 3.1. Participants and Procedures

An online survey was conducted via an online survey website (Credamo, https://www.credamo.com, accessed on 11 October 2021). Before the collection of formal questionnaire responses (Appendix A), we conducted a pre-survey and adjusted the items according to the results of the pre-survey. There are two main methods of sample collection: one is to collect data through the data mart of the Credamo questionnaire platform, and the other is to forward the questionnaire link on multiple social platforms to invite others to answer the questionnaire. The questionnaire could not be answered more than once by an individual; a respondent at a given IP address could only answer the questionnaire once. The questionnaire consisted of three parts. The first part asked the participants to answer a question which determined whether they participated in VBCs and determined the type of VBCs they participated in most often. The second part of the questionnaire contains four rating scales, which required participants to answer in accordance with the kind of VBC they were most familiar with. The third part of the questionnaire collected the demographic data of the participants. After reading the information describing the VBCs, participants were asked whether they participated in VBCs (including registration/browsing/posting/commenting/participating in interactions within the community). Those who had not participated in a VBC were not the subjects of this study. Data was collected from 11 October to 27 November 2021. A total of 380 questionnaires were distributed. After screening the validity of the questionnaire responses, 293 valid responses were collected; the recovery rate of the effective questionnaire responses was 77%. Sample characteristics are presented in Table 1.

### 3.2. Measures

Intercustomer social support: Intercustomer social support consists of two dimensions—informational support and emotional support. Measurements for informational and emotional support were adopted from the studies of Liang et al. [43] and Zhu et al. [23]. A total of eight items were used to measure intercustomer social support: four items were used to measure informational support (e.g., “In the community, some customers would offer suggestions when I needed help”); four items were used to measure emotional support (e.g., “When I faced with difficulties, some customers on the community are on my side with me”).

Customer engagement behaviors: CEBs have two dimensions—community-oriented engagement behaviors and customer-oriented engagement behaviors. Community-oriented engagement behaviors comprise three behavior types: cooperation, feedback, and compliance. Customer-oriented engagement behaviors comprise two behavior types: positive word-of-mouth and helping others. These two measurement scales were adopted by Verleye et al. [17], Han et al. [45], and Li et al. [72]. According to the results of the pre-survey, this study eliminated three items whose factor load did not meet the standard [73]. Therefore, a total of twelve items were used to measure the CEBs, with six items for measuring community-oriented engagement behaviors (e.g., “I usually cooperate with the brand community workers”) and six items for measuring customer-oriented engagement behaviors (e.g., “I promote the positive aspects of the brand community to others”).

Self-efficacy: The self-efficacy scale was adopted by Zhihong et al. [74] and includes four items (e.g., “I have the confidence to use the various functions of the community in the absence of guidance”).

Interdependent self-construal: The interdependent self-construal scale was adopted from Lee et al. [32] and includes five items (e.g., “It is important to me to respect decisions made by the group in community”).

All the subscale items were measured using seven-point Likert scales, ranging from 1 (strongly disagree) to 7 (strongly agree).

### 3.3. Data Analysis

SPSS and AMOS were used to analyze the data collected. Before testing the hypothesis, a confirming factor analysis (CFA) was used to examine the fit of the overall model; reliability and validity were calculated to assess the stability and effectiveness of the subscales. The structural equation model (SEM) was used to determine the effects of intercustomer social support on CEBs and to determine the mediating role of self-efficacy in the above relationships via AMOS. A hierarchical regression analysis was used to examine the moderating role of interdependent self-construal via SPSS 23.0.

### 3.4. Common Method Bias

This study controlled the common method bias by obtaining data from different sources and using an anonymous method; the Harman’s single factor test was used to test the common method bias [75]. This study conducts an exploratory factor analysis and examines the unrotated factor solution. There are six factors with character roots greater than 1, and the first factor explains 32.42% of the total variation, which is less than the critical value of 50%. This suggests that there is no serious common method bias that exists.

## 4. Results

### 4.1. Measurement of Model

Firstly, the results of the CFA showed that the degree of model fit is acceptable (*χ^2^*/*df* = 1.575, RMSEA = 0.044, GFI = 0.881, IFI = 0.956, TLI = 0.950, CFI = 0.955) [76]. Secondly, regarding the reliability analysis presented in Table 2, the Cronbach’s alpha values of all subscales were higher than 0.8, and the composite reliability (CR) values of all subscales were higher than 0.8, indicating acceptable internal reliability (as suggested by Bagozzi and Yi [77]). In addition, the factor-loading values of all measurement items were higher than 0.6, exceeding the acceptable value of 0.5 [73]. Finally, convergent validity and discriminant validity were analyzed to examine construct validity. As shown in Table 2, the average variance extracted (AVE) values of all subscales were higher than 0.5, suggesting convergent validity [77]. As shown in Table 3, the square root of the AVE of all the subscales was higher than the inter-construct correlations, supporting the discriminant validity [78].

### 4.2. Hypothesis Testing

**Figure 2:**
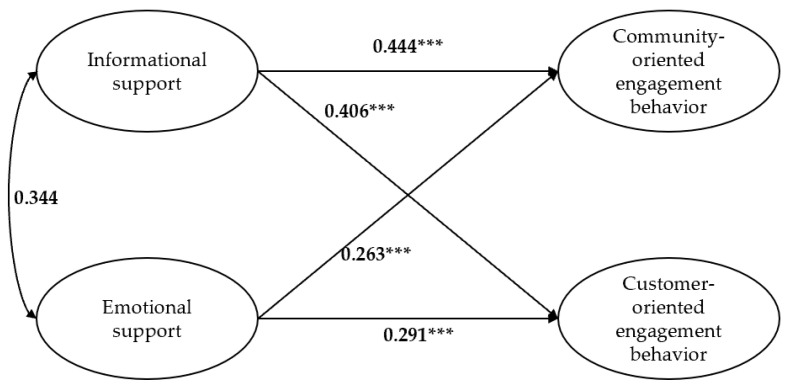
Direct effects of informational/emotional support on community-oriented/customer-oriented engagement behavior. Note: N = 293, *** p < 0.001.

The effects of two dimensions of intercustomer social support on two dimensions of customer engagement behaviors were examined through the SEM, using the maximum likelihood method. The results show that the direct-effects structural model provided an acceptable fit to the data (*χ^2^/df* = 1.952, RMSEA = 0.057, GFI = 0.898, IFI = 0.945, TLI = 0.936, CFI = 0.945) [76]. As shown in Figure 2, informational support has a positive effect on community-oriented engagement behavior (*β* = 0.444, *p* < 0.001); informational support has a positive effect on customer-oriented engagement behavior (*β* = 0.406, *p* < 0.001); emotional support has a positive effect on community-oriented engagement behavior (*β* = 0.263, *p* < 0.001); emotional support has a positive effect on customer-oriented engagement behavior (*β* = 0.291, *p* < 0.001). H1a, H1b, H2a, and H2b were thus supported.

**Figure 3:**
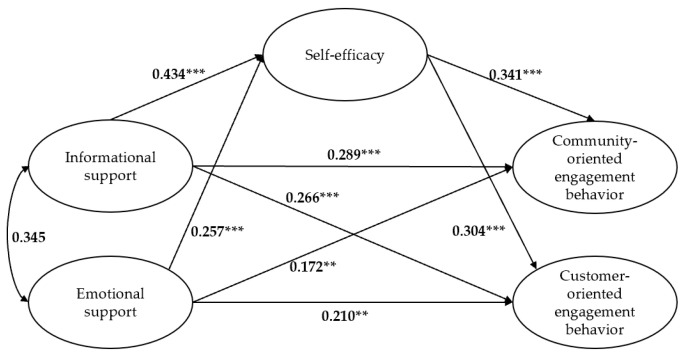
Mediation model of effects of informational support, emotional support, self-efficacy, community-oriented engagement behavior, and customer-oriented engagement behavior. Note: N = 293, *** p < 0.001; ** p < 0.01.

For testing the mediating role of self-efficacy, we used SEM with the maximum likelihood method and performed percentile bootstrapping and bias-corrected percentile bootstrapping at a 95% confidence interval with 5000 bootstrap samples. The confidence intervals of the lower and upper bounds were calculated to determine whether the indirect effects were significant according to the thresholds suggested by Preacher and Hayes [79]. The results show that the mediation effect structural model provided an acceptable fit to the data (*χ^2^*/*df* = 1.633, RMSEA = 0.047, GFI = 0.897, IFI = 0.957, TLI = 0.951, CFI = 0.957) [76]. As shown in Figure 3, informational support was found to positively affect self-efficacy (*β* = 0.434, *p* < 0.001); emotional support was found to positively affect self-efficacy (*β* = 0.257, *p* < 0.001); self-efficacy was found to positively affect community-oriented engagement behavior (*β* = 0.341, *p* < 0.001); self-efficacy was found to positively affect customer-oriented engagement behavior (*β* = 0.304, *p* < 0.001). The direct effect results of informational support on community-oriented engagement behavior (*β* = 0.289, *p* < 0.001), those of informational support on customer-oriented engagement behavior (*β* = 0.266, *p* < 0.001), those of emotional support on community-oriented engagement behavior (*β* = 0.172, *p* < 0.01), and those of emotional support on customer-oriented engagement behavior (*β* = 0.210, *p* < 0.01) were all statistically significant. In addition, the results of the bootstrap test presented in Table 4 show that the mediating effects for self-efficacy between informational support and community-oriented engagement behavior (*β* = 0.148, *p* < 0.01), between informational support and customer-oriented engagement behavior (*β* = 0.132, *p* < 0.01), between emotional support and community-oriented engagement behavior (*β* = 0.088, *p* < 0.05), and between emotional support and customer-oriented engagement behavior (*β* = 0.078, *p* < 0.05) were all statistically significant. H3a, H3b, H3c, and H3d were thus supported.

For testing the moderating role of interdependent self-construal, this study used hierarchical regression. All variables were standardized to reduce the potential effects of multicollinearity, as suggested by Cohen et al. [80]. The technique of least squares was used with the control variables entered, followed by the main effects and the interaction effects in the last step. As shown in Table 5, the interaction term (IS*ISC) in Model III explained significant variance beyond Model II (Δ*R*^2^ = 0.014, *p* < 0.05), and the interaction term (IS*ISC) in Model VI explained significant variance beyond Model V (Δ*R*^2^ = 0.019, *p* < 0.01). Furthermore, the interaction between informational support and interdependent self-construal (IS*ISC) had a positive effect on community-oriented engagement behavior (*β* = 0.131, *p* < 0.05), and the interaction between informational support and interdependent self-construal (IS*ISC) had a positive effect on customer-oriented engagement behavior (*β* = 0.156, *p* < 0.01), supporting H4a and H5a.

As shown in Table 6, the interaction term (ES*ISC) in Model IX did not explain significant variance beyond Model VIII (Δ*R*^2^ = 0.000, *p* > 0.05). Furthermore, the coefficient of the interaction between emotional support and interdependent self-construal (ES*ISC) was not significant when the dependent variable was community-oriented engagement behavior, failing to support H4b. The interaction term (ES*ISC) in Model XII explained significant variance beyond Model XI (Δ*R*^2^ = 0.022, *p* < 0.01). Furthermore, the interaction between emotional support and interdependent self-construal (ES*ISC) had a positive effect on customer-oriented engagement behavior (*β* = 0.164, *p* < 0.01), supporting H5b.

## 5. Discussion

### 5.1. Theoretical Implications

First, this study developed a model that integrated intercustomer social support and CEBs, examining the effects of intercustomer social support on CEBs. Although a previous study discussed the relationship between support from other customers and CEBs [17], this is the first study that extends the above relationships to the context of VBCs, which is a non-contact service scenario. In line with the study of Verely et al. [17], this study found that social support from other customers can encourage all forms of CEBs. Specifically, informational support and emotional support from other customers can enhance the degree of role readiness and increase the positive mood of customers [17,23,49], which encourages community-oriented engagement behaviors. Due to reciprocal motivation, customers who receive informational support and emotional support from other customers perform customer-oriented engagement behavior.

In addition, this study contributes to the literature on intercustomer social support by addressing a different form of intercustomer social support—namely, informational support. Distinctly from previous studies [25,41], this study considers the virtuality of VBCs [23]; that is, the support people receive from other customers in VBCs is intangible. Thus, building on existing social support research based on the online environment [23,43], this study takes informational support as a form of intercustomer social support. As a result, this study improves the scholarly understanding of intercustomer social support in VBCs.

Second, this study extends the findings of Bravo et al. [28] by discussing the presence of self-efficacy. Bravo et al. [28] demonstrated that self-efficacy positively affects CEBs, including WOM, feedback, content, and searching behaviors. Drawing on social cognitive theory [27], this study considered the influence of intercustomer social support on self-efficacy [40,41], and developed a more complete theoretical model that integrates intercustomer social support, self-efficacy, and CEBs. Thus, this study examined the mediating role of self-efficacy in the relationship between intercustomer social support and CEBs. The results show that informational support and emotional support positively affect community-oriented engagement behavior and customer-oriented engagement behavior through self-efficacy. This conclusion is consistent with the social cognitive theory, which explains that self-efficacy can influence an individual’s reaction to the environment, strongly dominating that individual’s behaviors [27,55]. Specifically, informational support and emotional support—as a vicarious experience factor and an emotional arousal factor, respectively—enhance customers’ self-efficacy [27,29], encouraging CEBs.

Third, this study expands the boundary conditions of the effects of intercustomer social support on CEBs by examining the moderating role of interdependent self-construal. The results show that interdependent self-construal positively moderates the relationship between informational support/emotional support and customer-oriented engagement behaviors; additionally, it positively moderates the relationship between informational support and community-oriented engagement behaviors. This conclusion is consistent with the self-construal theory [30], which points out that individuals with interdependent self-construal see themselves as part of an encompassing social relationship and their actions are influenced by the views and attitudes of others in the relationship. In addition, this finding confirms the view of Moses et al. [33], who explain that those with high levels of interdependent self-construal play an important role in fostering online communities through user-generated content. The higher the level of interdependent self-construal an individual has, the more they perceive social support [66]; this high perceived level of intercustomer social support encourages them to perform community-oriented and customer-oriented engagement behaviors. The moderating role of interdependent self-construal in the relationship between emotional support and community-oriented engagement behavior is not significant. Mathwick et al. [81] explained why this happens. Mathwick et al. [81] pointed out that, when people have limited knowledge of the products or brands of the communities they participate in, their primary motivation is often to obtain valuable information from the community, rather than to provide information that is valuable to the community. Emotional support deepens the emotional connection between customers in the group but does not increase individual brand knowledge or skills. Due to their ability constraints, customers with high levels of interdependent self-construal do not necessarily show higher levels of community-oriented engagement behavior than customers who have low levels of interdependent self-construal, even though they are perceived to provide high levels of emotional support by other customers.

### 5.2. Managerial Implications

This research yields some key implications for practitioners. First, practitioners need to re-examine their views on customer relationship management. That is, they must manage the relationship between the enterprise and the customers while paying attention to the healthy development of relationships among their customers. This study confirms that intercustomer social support not only directly affects CEBs, but also encourages CEBs through self-efficacy. Therefore, practitioners should pay attention to the page design and functional optimization of VBCs to promote intercustomer social support. They could divide special interactive segments according to regions, customer characteristics, products, or service types, and make as many functional segments as possible the platform for intercustomer social support on the basis of community settings. For informational support among customers, practitioners could optimize the retrieval function within the community, expand the channels for information provision and acquisition, and refine the information classification section to help with the correct docking between the information provider and the recipient. For emotional support among customers, practitioners can widely publicize the slogan “community family” in the community, calling on customers in the community to respect and care for each other. In addition, medals such as “Community Mutual Assistance”, “Social Talent”, and “Community Expert” can be awarded to those who provide social support among customers to increase the enthusiasm for intercustomer social support in the VBCs.

Second, this study demonstrated that interdependent self-construal can positively moderate the relationship between informational/emotional support and customer-oriented engagement behavior, and positively moderate the relationship between informational support and community-oriented behavior. Thus, practitioners should take measures to activate and enhance customers’ levels of interdependent self-construal. Specifically, practitioners can initiate individual interdependent self-construal from the perspective of culture. Using words such as “we”, “ours”, “everyone”, and “common” as much as possible when publicizing activities or issuing announcements in the community can enhance customers’ interdependent self-construal. In addition, practitioners can launch online collective activities that allow customers to cooperate with others to complete tasks, which can help enhance their sense of togetherness.

### 5.3. Limitations and Future Research Directions

This study has some limitations: First, there are different classifications of VBCs. For example, according to the criterion of who hosts them, there are two major types of VBCs—consumer-initiated communities and company-initiated communities [82]. Different types of VBCs have different operating mechanisms [1,82]. However, this study did not discuss the effects of intercustomer social support on CEBs for different types of VBCs. Therefore, future studies can examine the differences in the effects of intercustomer social support on CEBs in different types of VBCs. Second, the questionnaire is distributed via an online platform, and the people who participate in VBCs are mostly young and middle-aged in China. Future studies can consider expanding the number of elderly samples through the offline distribution of questionnaires and obtaining more samples from different countries. Third, this study uses a self-report method, and the data is cross-sectional data rather than time series data. Individuals’ cognition and behavior patterns change over time. Future studies can consider using time series data to measure the causal relationship between intercustomer social support and CEBs.

### 5.4. Conclusions

In conclusion, in the context of VBCs, this study examines the effects of intercustomer social support on CEBs and considers the mediating role of self-efficacy and the moderating role of interdependent self-construal in the above relationships. This study finds that informational support and emotional support both positively affect community-oriented engagement behaviors and customer-oriented engagement behaviors. Informational support and emotional support can also have positive effects on community-oriented engagement behaviors and customer-oriented engagement behaviors via self-efficacy. Moreover, interdependent self-construal plays a positive moderating role between informational support/emotional support and customer-oriented engagement behaviors and a positive moderating role between informational support and community-oriented engagement behaviors.

## Figures and Tables

**Table 1:** Sample description.

Demographics	Frequency	Percentage (%)
*Gender*		
Men	119	40.6
Women	174	59.4
*Age range*		
Under 20	17	5.8
From 21 to 30	149	50.9
From 31 to 40	102	34.8
Over 41	25	8.5
*Educational level*		
High school/technical secondary school and below	17	5.8
Associated degrees	57	19.5
Undergraduate degrees	172	58.7
Postgraduate degrees	47	16.0
*Occupation*		
Civil servant	12	4.1
Company manager	46	15.7
Ordinary employees of the enterprise	109	37.2
Student	102	34.8
Freelancer	10	3.4
Retired	6	2.0
Others	8	2.7
*Type of VBCs*		
Electronic product VBCs	100	34.1
Automobile VBCs	32	10.9
Cosmetics VBCs	82	28.0
Game VBCs	57	19.5
Others	22	7.5
Total	293	100

**Table 2:** Latent variables statistics.

Construct	Item	Loading	CR	AVE	Cronbach’s alpha
Informational support	IS1	0.695	0.853	0.593	0.852
	IS2	0.767			
	IS3	0.843			
	IS4	0.767			
Emotional support	ES1	0.787	0.851	0.588	0.850
	ES2	0.771			
	ES3	0.777			
	ES4	0.732			
Self-efficacy	SE1	0.772	0.883	0.654	0.882
	SE2	0.798			
	SE3	0.861			
	SE4	0.801			
Interdependent self-construal	ISC1	0.816	0.915	0.682	0.914
	ISC2	0.798			
	ISC3	0.812			
	ISC4	0.857			
	ISC5	0.845			
Community-oriented engagement behavior	COOEB1	0.661	0.890	0.576	0.889
	COOEB2	0.790			
	COOEB3	0.765			
	COOEB4	0.764			
	COOEB5	0.779			
	COOEB6	0.788			
Customer-oriented engagement behavior	CUOEB1	0.682	0.859	0.505	0.859
	CUOEB2	0.738			
	CUOEB3	0.664			
	CUOEB4	0.665			
	CUOEB5	0.741			
	CUOEB6	0.766			

Note: N = 293; IS—informational support, ES—emotional support, SE—self-efficacy, ISC—interdependent self-construal, COOEB—community-oriented engagement behavior, CUOEB—customer-oriented engagement behavior.

**Table 3:** Correlation of constructs and square root of AVE.

Variable	1	2	3	4	5	6
1 Informational support	0.770					
2 Emotional support	0.288 **	0.767				
3 Community-oriented engagement behavior	0.459 **	0.358 **	0.759			
4 Customer-oriented engagement behavior	0.413 **	0.360 **	0.444 **	0.711		
5 Self-efficacy	0.450 **	0.358 **	0.496 **	0.455 **	0.809	
6 Interdependent self-construal	0.237 **	0.220 **	0.320 **	0.328 **	0.269 **	0.826

Note: N = 293, ** *p* < 0.01.

**Table 4:** Standardized direct, indirect, and total effects of the mediation model.

	Point Estimate	SE	Z	Bias-Corrected 95%CI		Percentile 95%CI	
				Lower	Upper	Lower	Upper
*indirect effects*							
IS→SE→COOEB	0.148 **	0.052	2.846	0.066	0.274	0.056	0.258
IS→SE→CUOEB	0.132 **	0.049	2.694	0.055	0.251	0.046	0.236
ES→SE→COOEB	0.088 *	0.041	2.146	0.028	0.192	0.022	0.181
ES→SE→CUOEB	0.078 *	0.037	2.108	0.024	0.175	0.017	0.161
*direct effects*							
IS→COOEB	0.289 **	0.101	2.861	0.095	0.484	0.101	0.488
IS→CUOEB	0.266 **	0.104	2.558	0.063	0.471	0.063	0.471
ES→COOEB	0.172 *	0.083	2.072	0.020	0.345	0.013	0.337
ES→CUOEB	0.210 **	0.081	2.593	0.064	0.379	0.061	0.376
*total effects*							
IS→COOEB	0.437 ***	0.085	5.141	0.263	0.597	0.268	0.603
IS→CUOEB	0.398 ***	0.093	4.280	0.212	0.577	0.211	0.577
ES→COOEB	0.259 **	0.088	2.943	0.095	0.437	0.087	0.430
ES→CUOEB	0.289 **	0.088	3.284	0.117	0.460	0.119	0.463

Note: N = 293; IS—informational support; ES—emotional support; SE—self-efficacy; COOEB—community-oriented engagement behavior; CUOEB—customer-oriented engagement behavior. *** *p* < 0.001; ** *p* < 0.01; * *p* < 0.05.

**Table 5:** The result of the moderation of interdependent self-construal in the relationship between informational support and the two dimensions of CEBs.

Predictor Variables	Community-Oriented Engagement Behavior			Customer-Oriented Engagement Behavior		
	I	II	III	IV	V	VI
Gender	−0.059	−0.022	−0.015	−0.051	−0.014	−0.005
Age	0.019	−0.072	−0.069	0.116 *	0.033	0.037
Education	0.064	0.091	0.089	−0.008	0.019	0.016
Occupation	−0.094	−0.068	−0.066	−0.063	−0.043	−0.040
Community type	−0.033	−0.039	−0.039	−0.079	−0.090	−0.090
IS		0.403 ***	0.413 ***		0.337 ***	0.348 ***
ISC		0.241 ***	0.296 ***		0.254 ***	0.320 ***
IS*ISC			0.131 *			0.156 **
Δ*R*^2^	0.024	0.256	0.014	0.031	0.209	0.019
F	1.403	50.630 ***	5.508 *	1.824	39.252 ***	7.444 **
TOL	0.945–0.983	0.903–0.960	0.753–0.960	0.945–0.983	0.903–0.960	0.753–0.960
VIF	1.017–1.058	1.042–1.107	1.042–1.328	1.017–1.058	1.042–1.107	1.042–1.328

Note: N = 293; IS—informational support; ISC—interdependent self-construal. *** *p* < 0.001; ** *p* < 0.01; * *p* < 0.05.

**Table 6:** The result of the moderation of interdependent self-construal in the relationship between emotional support and the two dimensions of CEBs.

Predictor Variables	Community-Oriented Engagement Behavior			Customer-Oriented Engagement Behavior		
	VII	VIII	IX	X	XI	XII
Gender	−0.059	0.018	0.018	−0.051	0.028	0.035
Age	0.019	−0.013	−0.013	0.116*	0.086	0.078
Education	0.064	0.074	0.074	−0.008	0.002	0.004
Occupation	−0.094	−0.083	−0.083	−0.063	−0.051	−0.044
Community type	−0.033	−0.094	−0.094	−0.079	−0.140 **	−0.146 **
ES		0.298 ***	0.299 ***		0.311 ***	0.347 ***
ISC		0.273 ***	0.275 ***		0.268 ***	0.320 ***
ES*ISC			0.006			0.164 **
Δ*R*^2^	0.024	0.189	0.000	0.031	0.195	0.022
F	1.403 *	34.316 ***	0.012	1.824	35.967 ***	8.339 **
TOL	0.945–0.983	0.914–0.964	0.822–0.962	0.945–0.983	0.914–0.964	0.822–0.962
VIF	1.017–1.058	1.037–1.095	1.040–1.217	1.017–1.058	1.037–1.095	1.040–1.217

Note: N = 293; ES—emotional support; ISC—interdependent self-construal. *** *p* < 0.001; ** *p* < 0.01; * *p* < 0.05.

## Data Availability

Not applicable.

## Appendix A

Questionnaire on Customer Engagement Behavior in Virtual Brand Communities

Dear Madam/Sir:

Hello! Thank you for taking the time to fill out this questionnaire. This is an academic questionnaire about customer engagement behaviors in virtual brand communities. This questionnaire is in the form of an anonymous survey. The data collected is only used for academic research. The information you fill in will be strictly confidential. Please rest assured. There is no right or wrong choice in the questionnaire, please choose according to your actual thoughts.

Part I:

Please read the questionnaire instructions before answering the questionnaire.

Questionnaire Description
A virtual brand community is a virtual platform for users to interact and communicate around a certain brand on the network. Including but not limited to official forums such as Xiaomi Community, Club of Huawei, VIVO Community, OPPO Community, Lenovo Community, Weifeng Network, Club Canon, Lancome Community, as well as official Weibo, WeChat official account, and so on established around a certain brand.

1. Have you ever participated in a virtual brand community? (Including registration/browsing/posting/commenting/participating in interaction within the community):

□ Yes

□ No

Please choose a virtual brand community that you are most familiar with and continue to answer the questions.

2. Which category of virtual brand communities do you participate in most often:

□ Electronic product virtual brand communities

□ Automobile virtual brand communities

□ Cosmetics virtual brand communities

□ Game virtual brand communities

□ Others

Part II: Academic Scale

Choose from the following statements based on the virtual brand community with which you are most familiar. (1 = Strongly Disagree, 2 = Disagree, 3 = A Little Disagree, 4 = Neutral, 5 = A Little Agree, 6 = Agree, 7 = Strongly Agree)

(1) Indicate the extent to which you agree/disagree with the following statements.

		Strongly Disagree	Neutral	Strongly Agree
1	In the community, some customers would offer suggestions when I needed help.	1 2 3 4 5 6 7		
2	In the community, some customers gave me information to help me overcome the problem.	1 2 3 4 5 6 7		
3	In the community, some customers helped me discover the course and provided me with suggestions to solve the problem.	1 2 3 4 5 6 7		
4	In the community, some customers told me the way to solve the problem.	1 2 3 4 5 6 7		
5	When I am faced with difficulties, some customers on the community are on my side with me.	1 2 3 4 5 6 7		
6	When I am faced with difficulties, some customers on the community comforted and encouraged me.	1 2 3 4 5 6 7		
7	When I am faced with difficulties, some customers on the community listened to me talk about my private feelings.	1 2 3 4 5 6 7		
8	Some customers on the community expressed interest and concern in my well-being.	1 2 3 4 5 6 7		

(2) Indicate the extent to which you agree/disagree with the following statements.

		Strongly Disagree	Neutral	Strongly Agree
9	In order to receive the service smoothly, I do some necessary things.	1 2 3 4 5 6 7		
10	In order to receive the service smoothly, I do what the brand community requires.	1 2 3 4 5 6 7		
11	In order to receive the service smoothly, I do what the brand community expects of me.	1 2 3 4 5 6 7		
12	I usually cooperate with the brand community workers.	1 2 3 4 5 6 7		
13	If the brand’s community staff makes a mistake, I will give feedback.	1 2 3 4 5 6 7		
14	I provide constructive suggestions to the brand community to improve its services.	1 2 3 4 5 6 7		
15	I recommend the brand community to people interested in the brand.	1 2 3 4 5 6 7		
16	I recommend this brand community to my family and friends.	1 2 3 4 5 6 7		
17	I promote the positive aspects of the brand community to others.	1 2 3 4 5 6 7		
18	I help other customers understand the brand better.	1 2 3 4 5 6 7		
19	I help other customer if necessary.	1 2 3 4 5 6 7		
20	I explain to other customers which services are provided by the brand community.	1 2 3 4 5 6 7		

(3) Indicate the extent to which you agree/disagree with the following statements.

		Strongly Disagree	Neutral	Strongly Agree
21	I have the confidence to use the various functions of the community in the absence of guidance.	1 2 3 4 5 6 7		
22	I am confident that I can provide valuable knowledge.	1 2 3 4 5 6 7		
23	I have the necessary skills, experience, and insights to provide valuable knowledge.	1 2 3 4 5 6 7		
24	I have the confidence to respond or comment on the information and articles from other members of the community.	1 2 3 4 5 6 7		

(4) Indicate the extent to which you agree/disagree with the following statements.

		Strongly Disagree	Neutral	Strongly Agree
25	My happiness depends on the happiness of my fellows in this community.	1 2 3 4 5 6 7		
26	I will sacrifice my self-interest for the benefit of the group I am in.	1 2 3 4 5 6 7		
27	It is important for me to maintain harmony with members of the community.	1 2 3 4 5 6 7		
28	I often have the feeling that my relationships with others are more important than my own accomplishments.	1 2 3 4 5 6 7		
29	It is important to me to respect decisions made by the group in the community.	1 2 3 4 5 6 7		

Part III: Basic Personal Information

1. Gender

□ Men □ Women

2. Age

□ Under 20 □ 21–30 □ 31–40 □ Over 41

3. Educational level

□ High school/technical secondary school and below
□ Associated degrees
□ Undergraduate degrees
□ Postgraduate degrees

4. Occupation

□ Civil servant □ Company manager □ Ordinary employees of the enterprise
□ Student □ Freelancer □ Retired □ Others
Thank you for completing this survey.
